# A multi-institutional and case-matched control study on treatment outcomes of consolidative radiotherapy after a full course of R-CHOP compared with R-CHOP alone in Stage I–II diffuse large B-cell lymphoma (KROG 17-02)

**DOI:** 10.1093/jrr/rrz043

**Published:** 2019-06-28

**Authors:** Mi Joo Chung, Won Kyung Cho, Dongryul Oh, Keun-Yong Eom, Jin Hee Kim, Woo Chul Kim, Jong Hoon Lee

**Affiliations:** 1 Department of Radiation Oncology, Kyung Hee University Hospital at Gangdong, College of Medicine, Kyung Hee University, Seoul, Korea; 2 Department of Radiation Oncology, Samsung Medical Center, Sungkyunkwan University School of Medicine, Seoul, Korea; 3 Department of Radiation Oncology, Seoul National University Bundang Hospital, Seongnam, Korea; 4 Department of Radiation Oncology, Dongsan Medical Center, Keimyung University School of Medicine, Daegu, Korea; 5 Department of Radiation Oncology, Inha University Hospital, Inha University of Medicine, Inchon, Korea; 6 Department of Radiation Oncology, St Vincent’s Hospital, College of Medicine, The Catholic University of Korea, 442-723, 93-6, Ji-dong, Paldal-gu, Suwon, Kyeonggi-do, Republic of Korea

**Keywords:** consolidative radiation therapy, R-CHOP, diffuse large B-cell lymphoma

## Abstract

We compared treatment outcomes between rituximab, cyclophosphamide, doxorubicin, vincristine, and prednisone (R-CHOP) chemotherapy alone with R-CHOP followed by consolidative radiation therapy (RT) in diffuse large B-cell lymphoma (DLBCL). We analyzed 404 patients with Stage I–II DLBCL who received six to eight cycles of R-CHOP and achieved a good response after a full course of chemotherapy. Propensity-score matching was used to assess the role of consolidative RT. The R-CHOP alone group (*n* = 184) was matched in a 1:2 ratio with the R-CHOP plus RT group (*n* = 92). Twenty-four (13.0%) of 184 patients receiving R-CHOP alone and 8 (8.7%) of 92 patients receiving R-CHOP plus RT had bulky diseases (>7.5 cm). A Deauville score of 1–2 was achieved for 159 (86.4%) of 184 patients receiving R-CHOP alone and 84 (91.3%) of 92 patients receiving R-CHOP plus RT. After a median follow-up time of 42 months, the recurrence-free survival (RFS) rate (86.7% vs 93.0%, *P* = 0.464) and overall survival rate (88.3% vs 95.1%, *P* = 0.295) at 5 years did not differ significantly between the R-CHOP alone and R-CHOP plus RT arms. In the additional multivariate analyses, large tumor size (>7.5 cm) was significantly associated with decreased RFS (hazard ratio, 2.368 and confidence interval, 1.837–6.697; *P* = 0.048). Consolidative radiation was not a significant factor for RFS (*P* = 0.563). Tumor size was a significant factor for RFS in the rituximab era. The outcome of omitting consolidative RT for good responders after six to eight cycles of R-CHOP chemotherapy was acceptable in early-stage DLBCL without a bulky disease.

## INTRODUCTION

Diffuse large B-cell lymphoma (DLBCL) is the most common lymphoid neoplasm in adults [1, 2]. Over the past two decades, combination chemotherapy consisting of cyclophosphamide, doxorubicin, vincristine and prednisone (CHOP) has become a standard regimen for DLBCL [3]. The role of consolidative radiation therapy (RT) after CHOP remains controversial. Results of reported prospective trials for the significance of RT after full-course CHOP chemotherapy have been conflicting in patients with DLBCL [4–8].

The East Coast Oncology Group (ECOG) 1484 trial has shown an improved failure-free survival in the RT arm after complete response to eight cycles of CHOP, supporting the role of RT even after good response to full-course chemotherapy [6]. On the other hand, the Groupe d’Etude des Lymphomes de l’Adulte (GELA) LNH 93-4 trial has shown no survival benefit of RT after four cycles of CHOP in elderly patients with Stage I–II aggressive lymphoma [4]. The Southwest Oncology Group (SWOG) 8736 trial has shown an improved overall survival at 5 years after three cycles of CHOP followed by RT than that with eight cycles of CHOP [7]. However, long-term follow-up data showed no significant difference in overall survival between the two arms.

Rituximab is a monoclonal antibody against protein CD20, which is primarily found on the surface of B-cells in the immune system. In the advent of rituximab (R), R-CHOP has led to a significant improvement in overall survival in elderly patients with DLBCL [9–11]. Whether consolidative RT after R-CHOP is still indicated or not remains unclear. The RICOVER-60 trial was the first prospective trial that assessed the role of RT in bulky (>7.5 cm) disease in elderly patients with DLBCL after R-CHOP chemotherapy [5]. The R-CHOP only arm was found to have a significantly higher recurrence rate than the R-CHOP followed by RT arm. However, in the modern era, the addition of consolidative RT has been gradually decreasing [11]. Thus, the objective of this multicenter study was to compare treatment outcomes between R-CHOP chemotherapy alone and R-CHOP followed by consolidative RT in DLBCL in order to identify the role of consolidative RT for patients with DLBCL.

## MATERIALS AND METHODS

### Patients

We retrospectively enrolled 404 patients with DLBCL. Of these, 312 patients received R-CHOP only and 92 patients received R-CHOP followed by RT at the sole discretion of the treating medical oncologists in five tertiary institutions from January 2010 to December 2015. Inclusion criteria for this study were: (i) histologically proven DLBCL of clinical Stage I to II; (ii) ECOG performance status 0–2; (iii) initial treatment with six to eight cycles of R-CHOP chemotherapy; and (iv) good response (Deauville scale 1–3) in ^18^F-fluoro-deoxy-glucose Positron Emission Tomography (PET)-CT after R-CHOP. Patients with primary CNS lymphoma were excluded from this study. Institutional Review Board approval was obtained at each participating center before enrolling patients (VC17RESI0046).

Chemotherapy, RT, pathology, and follow-up records of each patient were reviewed using a data management program. Clinical stage for DLBCL was determined according to the Ann Arbor staging system. Initial work-ups included physical examination, evaluation of ECOG performance status, and constitutional symptoms such as fever, weight loss, and night sweats. Laboratory assessments included complete blood counts, blood chemistry, serum lactate dehydrogenase (LDH), bone marrow biopsy, and tissue biopsy. Imaging studies included neck, chest, and abdominopelvic CT as well as PET-CT before and 2–3 months after chemotherapy. We assessed the response to R-CHOP according to the Deauville five-point scale using PET-CT [12, 13]. International prognostic index (IPI) scores were checked based on the patient’s age, stage of disease, serum LDH level, ECOG performance status, and the number of extranodal sites.

### Treatment

All patients received six to eight cycles of R-CHOP (intravenous rituximab, 375 mg/m^2^; cyclophosphamide, 750 mg/m^2^; doxorubicin, 50 mg/m^2^; vincristine, 1.4 mg/m^2^; and oral prednisolone, 100 mg). Consolidative RT was administered at a median dose of 36 Gy (range, 26–56 Gy) at 1.8–2 Gy per fraction 1–2 months after R-CHOP.

### Study design and propensity-score matching

**Fig. 1.**
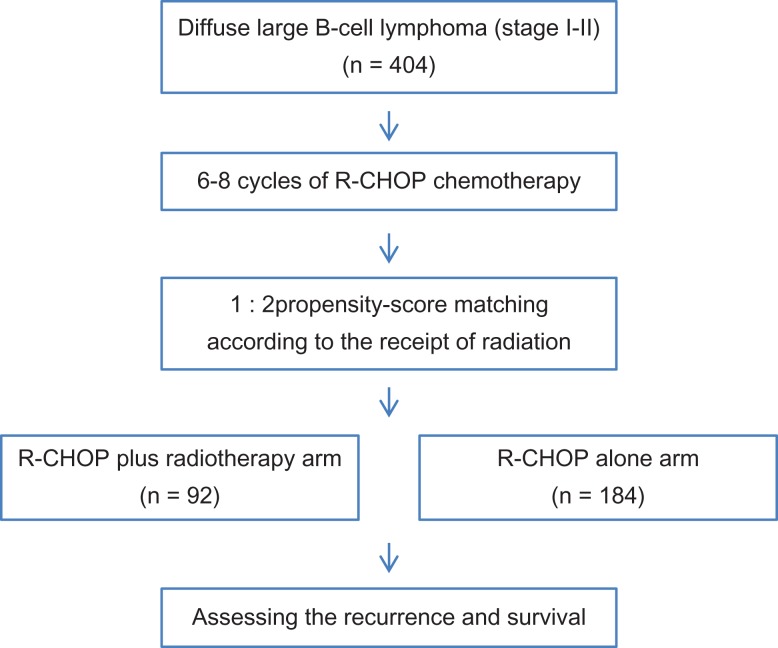
Flow chart showing patient enrollment, matching and assessment.

To accurately compare treatment outcomes between R-CHOP alone and R-CHOP followed by consolidative RT arms, we performed propensity-score matching for enrolled patients and analyzed their recurrence and survival rates after matching (Fig. 1). Propensity scores were calculated using a multivariate logistic-regression model based on the following variables: age, ECOG performance status, clinical stage, tumor size, LDH level, IPI score, Deauville score, and extranodal disease. A total of 92 patients in the R-CHOP plus RT arm and 184 patients in the R-CHOP alone arm were matched at a 1:2 ratio. The matching model was well calibrated (Hosmer–Lemeshow test, *P* = 0.931) with reasonable discrimination (c-index = 0.652).

### Statistical analyses

Patient characteristics were compared using the χ^2^ test for categorical variables and the *t*-test for continuous variables. Primary end points of this study were recurrence-free survival (RFS) and overall survival (OS). Secondary end points were patterns of relapse such as locoregional recurrence and distant metastasis. Kaplan–Meier analysis with the log-rank test was used for the univariate survival analysis. To evaluate prognostic factors related to recurrence and survival, multivariate analysis was performed with the Cox proportional hazard method. A *P*-value of less than 0.05 by two-tailed tests was considered to be statistically significant. All statistical analyses were performed using R software version 2.15 (Alcatel-Lucent, Murray Hill, NJ, USA) and SPSS software version 12.0 for Windows (SPSS Inc., Chicago, IL, USA).

## RESULTS

A total of 276 patients after propensity-score matching were finally analyzed. The median age was 54 years (range, 20–83 years) for the entire cohort. The median tumor size was 4 cm (range, 1–22.7 cm). Patient characteristics are summarized in **Table 1**. Patient age (*P* = 0.649), ECOG performance status (*P* = 0.147), clinical stage (*P* = 0.932), tumor size (*P* = 0.288), LDH level (*P* = 0.796), IPI score (*P* = 0.820) and Deauville score (*P* = 0.238) did not differ significantly between the R-CHOP alone and the R-CHOP plus RT groups. Extranodal disease (*P* = 0.001) did differ significantly between these two groups. Radiation treatment details are shown in **Table 2**. RT was delivered using 3D RT (*n* = 33) or intensity-modulated RT (*n* = 59). Of the 92 patients, 61 (66.3%) received involved-site RT and 31 (33.7%) received involved-field RT.

**Table 1.** Patient characteristics

Characteristic—No. (%)	R-CHOP (*n* = 184)	R-CHOP + RT (*n* = 92)	*P*-value
Age, year			0.649
≤60	123 (66.8)	64 (69.6)	
>60	61 (33.2)	28 (30.4)	
ECOG performance status			0.147
0	89 (48.4)	55 (59.8)	
1	89 (48.4)	36 (39.1)	
2	6 (3.2)	1 (1.1)	
Clinical stage			0.932
I	83 (45.1)	41 (44.6)	
II	101 (54.9)	51 (55.4)	
Tumor size, cm			0.288
≤7.5	160 (87.0)	84 (91.3)	
>7.5	24 (13.0)	8 (8.7)	
Lactate dehydrogenase, IU/l			0.796
≤230 (normal)	22 (12.0)	12 (13.0)	
>230 (elevated)	162 (88.0)	80 (87.0)	
IPI score			0.820
0–2 (low to low intermediate)	177 (96.2%)	89 (96.7%)	
3–4(high intermediate to high)	7 (3.8%)	3 (3.3%)	
Deauville score			0.238
1–2	159 (86.4%)	84 (91.3%)	
3	25 (13.6%)	8 (8.7%)	
Extranodal disease			0.001
No	63 (34.2%)	56 (60.9%)	
Yes	121 (65.8%)	36 (39.1%)	

ECOG = Eastern Cooperative Oncology Group, IPI = international prognostic index, R-CHOP = Rituximab, Cyclophosphamide, Doxorubicin, Vincristine and Prednisolone, RT = radiation therapy.

**Table 2.** Radiation treatment details

Characteristic—No. (%)	Radiotherapy arm (*n* = 92)
Radiation dose, Gy	
≤36	70 (76.1)
>36	22 (23.9)
Radiation technique	
Three-dimensional	33 (35.9)
Intensity-modulated	59 (64.1)
Radiation field	
Involved-site	61 (66.3)
Involved-field	31 (33.7)
Radiation duration, weeks	
≤4	70 (76.1)
>4	22 (23.9)

**Fig. 2.**
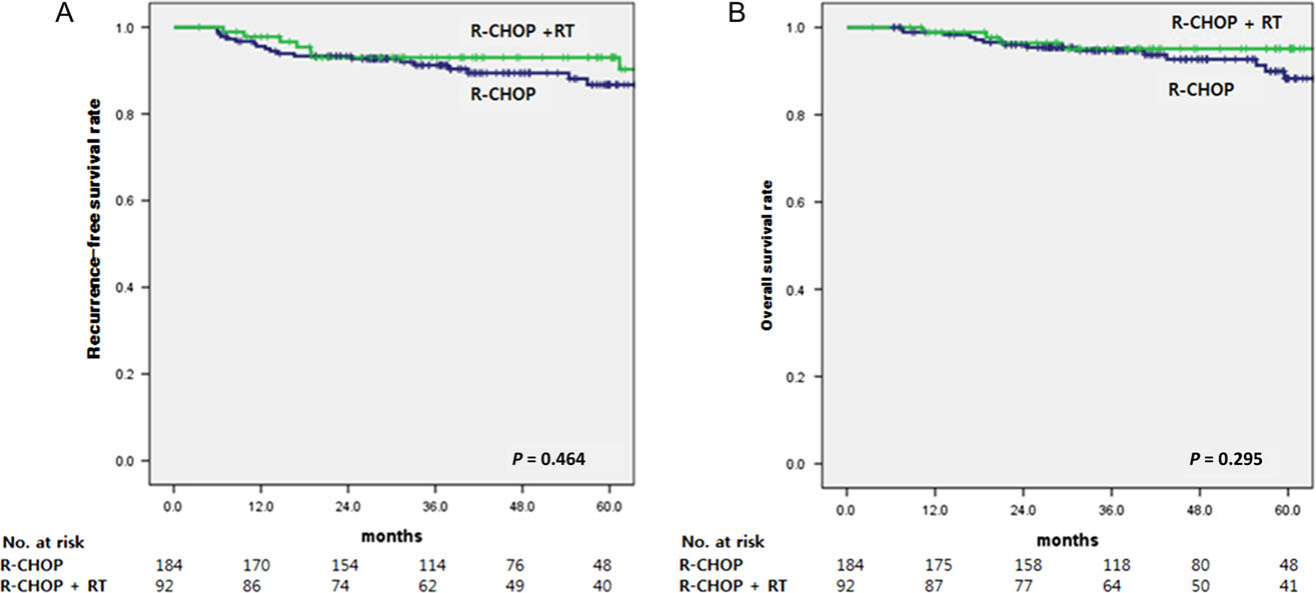
Recurrence-free survival rate (**A**) and overall survival rate (**B**) according to the receipt of radiotherapy after R-CHOP chemotherapy.

**Fig. 3.**
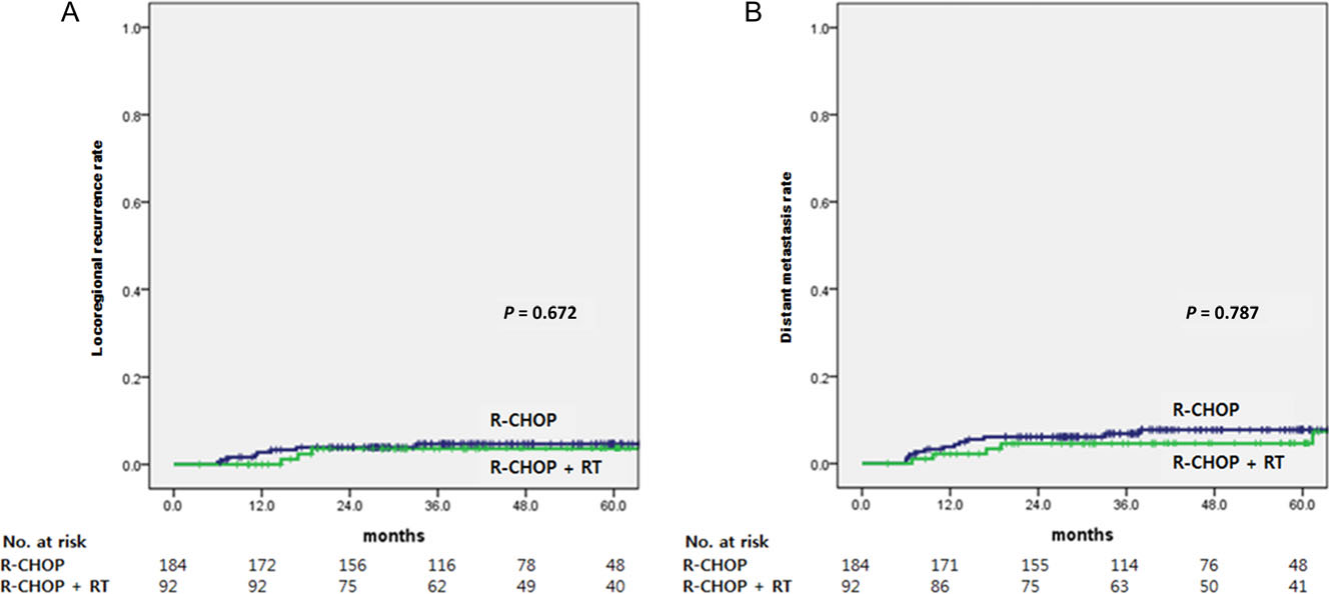
Locoregional recurrence rate (**A**) and distant metastasis rate (**B**) according to the receipt of radiotherapy after R-CHOP chemotherapy.

After a median follow-up time of 42 months, treatment failure including locoregional recurrence and distant metastasis was seen in 23 patients. Locoregional recurrence occurred in 8 (4.3%) of 184 patients with R-CHOP alone and 3 (3.3%) of 92 patients with R-CHOP plus RT. For patients with a bulky disease, locoregional recurrence occurred in 3 (12.5%) of 24 patients receiving R-CHOP alone and 1 (12.5%) of 8 patients receiving R-CHOP plus RT. Distant metastasis occurred in 13 (7.1%) patients in the R-CHOP alone arm and 7 (7.6%) patients in the R-CHOP plus RT arm. Eight patients had both locoregional and distant failures. Five-year RFS rates for the R-CHOP alone and R-CHOP plus RT arms were 86.7 % and 93.0%, respectively. Five-year OS rates for the R-CHOP alone and R-CHOP plus RT arms were 88.3% and 95.1%, respectively (Fig. 2). The difference in RFS (*P* = 0.464) or OS (*P* = 0.295) between the two arms did not reach statistical significance, despite the visual separation of the survival curves. Locoregional recurrence rate (4.7% vs 3.6%, *P* = 0.672) or distant metastasis rate (7.8% vs 7.3%, *p* = 0.787) at 5 years did not differ significantly between the R-CHOP alone and R-CHOP plus RT arms either (Fig. 3). In the R-CHOP plus RT arm, radiation dose (≤36 Gy vs >36 Gy) was not a significant factor for RFS (*P* = 0.356) or OS (*P* = 0.524).

In the univariate analysis, large tumor size (>7.5 cm) was significantly associated with decreased RFS (*P* = 0.035), and low to low-intermediate risk was significantly associated with improved OS (*P* = 0.041). In the additional multivariate analyses, tumor size was a significant factor for RFS (hazard ratio, 2.368 and confidence interval, 1.837–6.697; *P* = 0.048). Patient age, clinical stage, IPI score, consolidative radiation, Deauville score, and extranodal disease were not significant factors for RFS or OS (**Table 3**). In the R-CHOP plus RT arm, the radiation dose (≤36 Gy vs >36 Gy) was not a significant factor for RFS (*P* = 0.356) or OS (*P* = 0.524).

**Table 3.** Univariate and multivariate analyses of prognostic factors for recurrence-free survival and overall survival

	Recurrence-free survival				Overall survival			
Factors	5-year rate (%)	Univariate (*P*)	Hazard ratio (95% CI)	Multivariate (*P*)	5-year rate (%)	Univariate (*P*)	Hazard ratio (95% CI)	Multivariate (*P*)
Age, year		0.087		0.092		0.316		0.666
≤60	93.2		Referent		91.8		Referent	
>60	84.7		1.880 (0.902–3.919)		89.4		1.236 (0.472–3.242)	
Clinical stage		0.378		0.458		0.590		0.545
I	91.6		Referent		90.1		Referent	
II	87.2		1.344 (0.616–2.928)		91.7		0.760 (0.312–1.851)	
Tumor size, cm		0.035		0.048		0.954		0.749
≤7.5	89.6		Referent		90.4		Referent	
>7.5	85.3		2.368 (0.837–6.697)		96.9		1.284 (0.277–5.951)	
IPI score		0.148		0.378		0.041		0.060
Low to low intermediate	90.0		Referent		91.9		Referent	
High intermediate to high	67.5		2.032 (0.421–9.821)		65.6		4.079 (0.943–17.638)	
Consolidative radiation		0.464		0.563		0.295		0.407
No	86.7		Referent		88.3		Referent	
Yes	93.0		0.782 (0.340–1.801)		95.1		0.640 (0.223–1.836)	
Deauville score		0.723		0.563		0.366		0.343
1–2	88.9		Referent		91.6		Referent	
3	90.0		1.380 (0.463–4.116)		86.1		1.791 (0.537–5.976)	
Extranodal disease		0.779		0.479		0.963		0.570
No	88.1		Referent		91.2		Referent	
Yes	90.2		0.753 (0.343–1.653)		90.2		0.758 (0.292–1.972)	

ECOG = Eastern Cooperative Oncology Group, IPI = international prognostic index, R-CHOP = Rituximab, Cyclophosphamide, Doxorubicin, Vincristine and Prednisolone, HR = hazard ratio.

## DISCUSSION

Since 1980, localized non-Hodgkin’s lymphoma has usually been treated with CHOP with or without RT. Full-course CHOP or abbreviated-course CHOP followed by RT is recommended for the disease control of DLBCL. They also obviate the need for surgical staging in lymphoma [14]. RT is usually effective for disease confined to a treatment field. It permits reduction in the total dose of doxorubicin, which involves cardiotoxicity. This is a potential advantage for elderly patients with reduced myocardial reserve [15]. However, the role of consolidative RT has been uncertain, especially in the era of rituximab [16].

Randomized trials from the pre-rituximab era have shown that RT is valuable not only in reducing the number of courses of CHOP, and decreasing the toxicity, but also in improving RFS and OS over eight cycles of CHOP alone [6, 7]. In the SWOG 8736 trial, the full-course chemotherapy (eight cycles of CHOP) arm had significantly more frequent and more severe toxic reactions than the abbreviated chemotherapy (three cycles of CHOP) followed by RT arm [7]. The left ventricular function was also decreased in seven patients who received CHOP alone, whereas no cardiac events were recorded in the group receiving CHOP plus RT (*P* = 0.02).

In the rituximab era, several trials for R-CHOP chemotherapy alone in elderly patients with DLBCL showed drastic improvement in survival. This has brought about the question of whether consolidative RT after R-CHOP is still necessary or not [9–11]. No randomized controlled trial has been published to compare treatment outcomes between R-CHOP and R-CHOP plus consolidative RT for good responders with DLBCL after chemotherapy. Improved survival with the addition of consolidative RT following chemotherapy in DLBCL is consistent in subgroup analyses of recent prospective trials, including MInT (MabThera International Trial) and RICOVER-60 and several retrospective institutional studies, even after modern multiagent chemotherapy. The addition of consolidative RT improves outcomes, especially for patients with bulky disease or extranodal disease [11, 17–19]. Therefore, National Comprehensive Cancer Network guidelines still recommend three cycles of R-CHOP followed by RT for Stage I–II non-bulky disease or six cycles of R-CHOP with or without RT for all Stage I–II diseases [20].

Several studies have reported that six cycles of R-CHOP do not exclude the need for RT [8, 18]. Phan *et al.* have evaluated patients with DLBCL treated with R-CHOP or R-CHOP with RT [17]. Multivariate analysis showed that RT (*P* < 0.001) following R-CHOP administration was a significant prognostic factor for improved RFS. In the study of Marcheselli *et al.* [18], involved-field RT after R-CHOP yielded a significant event-free survival benefit, with a 66% reduction in the risk of death and/or disease progression (*P* < 0.05). Cox analysis, when adjusted for age, gender, stage, performance status, LDH, and disease bulk, confirmed the significant event-free survival benefit of radiation therapy. Vargo *et al.* analyzed the factors affecting treatment selection and the resulting survival outcomes in early-stage DLBCL in the modern era [20]. They suggested that abandonment of combined-modality therapy in favor of chemotherapy alone negatively affects patient survival. Vargo *et al.* included patients who were treated with not only R-CHOP but also CHOP chemotherapy. On the other hand, in our study, consolidative RT following R-CHOP was not associated with improved survival. Unlike the study by Vergo *et al.*, we only evaluated good responders with DLBCL treated with six to eight cycles of R-CHOP, not three cycles of R-CHOP. RT following abbreviated-course chemotherapy (i.e. three cycles), not full-course chemotherapy, might contribute more to local control and survival in Stage I–II and non-bulky DLBCL. Full-course R-CHOP over six cycles would obviate the additional impact of local disease control in the R-CHOP plus consolidative RT group. The minimum percentage of improved survival rate to achieve statistical significance in our cohort [experimental group (*n* = 92) : control group (*n* = 184)] was 0.15, when we set the 5-year survival rate of the control arm as 0.75, the significance level as 0.05, and the power as 0.8. Thus, a large-sized prospective study could be needed to verify the statistical significance of survival differences of <10% between the full-course R-CHOP with or without radiation arms. However, it is not easy to perform this large prospective randomized trial in a single nation.

Although the current study presents negative to use of consolidative RT for good responders after full-course R-CHOP, most previous studies in the rituximab era argued that consolidation RT improved outcome. Regarding the reason for the difference in outcomes between the present study and the previous reports, we evaluated good responders treated with full-course R-CHOP, and only 32 (11.6%) of 276 patients had bulky diseases (>7.5cm) in the present study. The study by Phan *et al.* evaluated 190 (40.5%) Stage I–II and 279 (59.5%) Stage III–IV patients with DLBCL after full-course R-CHOP and reported that the 5-year OS and progression-free survival (PFS) rates for Stage I and II disease treated with RT were 92% and 82%, respectively, whereas the OS and PFS rates for those not treated with RT were 73% and 68%, respectively; of the 190 patients with Stage I or II disease, RT was given to 103 patients (49 of the 103 had bulky disease) [17]. The great majority of patients in the current study were lower-risk patients than in the study of Phan *et al.*; our study included not only a lower rate of bulky disease, but also better PS, better IPI and better response (Deauville 1–2). Thus, the outcome for all patients was better than expected, and the advantage of consolidation RT diminished. In view of this, omitting consolidation RT is not recommended for patients, especially those with bulky disease.

The present multi-institutional retrospective analysis included 276 patients with DLBCL of Stage I–II who were treated with R-CHOP with or without RT. The decision for consolidative RT after full-course R-CHOP chemotherapy was at the discretion of the medical oncologists in this study. This in turn could represent a selection bias for the consolidative RT group. In the multidisciplinary team for lymphoma, the choice of consolidative RT in Stage I–II DLBCL should be carefully discussed among all physicians, including medical and radiation oncologists. In addition, radiation technique, dose, and fraction size varied according to each institutional policy [21, 22]. Such heterogeneity in the RT arm might be the reason for the lack of positive effect of RT after R-CHOP. Thus, we conducted 1:2 propensity score matching to correct heterogeneity of factors in this multi-institutional analysis. The results of this analysis should be interpreted with caution because of its retrospective nature.

## CONCLUSION

After the 3.5-year follow-up, the outcome of omitting consolidative RT for good responders after six to eight cycles of R-CHOP chemotherapy was acceptable in early-stage DLBCL without bulky disease. To verify our results, randomized multicenter trials that prospectively compare R-CHOP alone and R-CHOP with RT in good responders with DLBCL after full-course R-CHOP are now needed.
